# "Practical skills" – Positioning of the GMA committee for dentistry

**DOI:** 10.3205/zma001047

**Published:** 2016-08-15

**Authors:** Petra Scheutzel, Susanne Gerhard-Szép

**Affiliations:** 1 Universitätsklinikum Münster, Poliklinik für Prothetische Zahnmedizin & Biomaterialien, Münster, Germany; 2 Goethe-Universität, Carolinum Zahnärztliches Universitäts-Institut gGmbH, Poliklinik Zahnerhaltungskunde, Frankfurt am Main, Germany

**Keywords:** skills, practical skills, dental education, dental simulator, Dental Licensure Act

## Abstract

The GMA committee for dentistry of the German Society for Medical Education (GMA) considers its’ main purpose the representation and interconnection of all aspects of dentistry with and within the GMA. Teaching and assessing practical skills during training is traditionally of great importance in dental education. This is also reflected in the National Competence Based Catalogue of Learning Objectives for Dental Education (NKLZ). Practical skills are not comprised in a separate chapter as they are in the National Competence Based Catalogue of Learning Objectives for Medical Education (NKLM), but are considered in all sections of the NKLZ for the purpose of interdisciplinary patient- or disease-specific application, targeting the educational level of acting competency. The implementation of the associated joined interdisciplinary integrated educational concept has undoubtedly been a challenge for dental curriculum development against the backdrop of German Dental Licensure Act dating back to 1955.

## Zusammenfassung

Der Ausschuss Zahnmedizin der Gesellschaft für Medizinische Ausbildung (GMA) sieht seine Hauptaufgabe in der themenübergreifenden Vertretung und Vernetzung des Faches Zahnmedizin mit und innerhalb der GMA. In der Zahnmedizin ist die Vermittlung und Überprüfung praktischer Fertigkeiten traditionell von großer Bedeutung in der Ausbildung. Dies spiegelt sich auch im Nationalen Lernzielkatalog Zahnmedizin (NKLZ) wieder. Die praktischen Fertigkeiten sind nicht wie im Nationalen Lernzielkatalog Medizin (NKLM) in einem eigenen Kapitel zusammengefasst, sondern finden im Sinne einer fachübergreifenden patienten- bzw. erkrankungsspezifischen Anwendung in allen Abschnitten des NKLZ Berücksichtigung, und es wird häufig der Ausbildungslevel der Handlungskompetenz angestrebt. Die Umsetzung des damit verbundenen integrierten fachübergreifenden Ausbildungskonzeptes stellt für die zahnärztliche Curriculumsentwicklung vor dem Hintergrund einer seit 1955 gültigen Approbationsordnung zweifellos eine Herausforderung dar.

## 1. Introduction

### Duties and Objectives of the GMA Committee for Dentistry 

The GMA committee for dentistry holds, as well as the committee for veterinary medicine, a special status in comparison to the other committees insofar as it represents a discipline and not a single specific topic. 

Objective of the committee for dentistry is the linking of both dental teachers and dental learners to the German Society for Medical Education (GMA) and notably the interconnection between GMA and the Working Group for the Advancement of Dental Education (AKWLZ), which was institutionalized by the German Society for Dental and Oral Medicine (DGZMK) and the German Society for University Teachers of Dentistry and Oral Medicine (VHZMK). 

The GMA committee for dentistry, in close cooperation with the AKWLZ, deals with all matters concerning dental training, e.g. curriculum development, advancement and implementation of teaching, learning and assessment methods, the promotion of educational research and development and implementation of catalogues of learning objectives. The main focus lies with introducing and broadening knowledge gained in dental education into the interdisciplinary discourse with the other medical disciplines. 

## 2. Points of Contact with/Similarities to/Distinctions from the GMA Committee for Practical Skills

The GMA Committee for Practical Skills has developed from an initiative of German skills labs in medical education and consists of a network of these training facilities for practical medical skills in Germany. Consequently, the exchange of experience between the existing training facilities, support for the construction of new Skill Labs within the framework of medical training and research concerning the sharing of practical skills as part of Skill Labs form the basis of the committee’s work [https://gesellschaft-medizinische-ausbildung.org/ausschuesse/praktische-fertigkeiten.html]. Based on this, the GMA Committee for practical skills has participated significantly in the development of NKLM work package 14b, “Clinical and Practical Skills” [http://www.nklm.de/kataloge/nklm/lernziel/uebersichthttp://www.nklm.de/kataloge/nklm/lernziel/uebersicht]. The importance of imparting and verifying practical skills in medical education, which is reflected in this chapter of the NKLM, has only been initiated by the amendment of the German Medical Licensure Act for Physicians in 2002, increasing the importance of professional competencies in undergraduate medical education [1].

Contrary to this, practical skills always have been and still are accorded greater importance in undergraduate dental education. According to §1 of the still valid German Dental Licensure Act (Zahnärztliche Approbationsordnung - ZÄApprO) of 1955 [http://www.gesetze-im-internet.de/z_pro/BJNR000370955.html] which states:” For his profession the dentist is trained scientifically and practically”, both preclinical and clinical part of undergraduate dental curriculum contain several practical courses with training exercises on the dental simulator as well as laboratory courses and patient treatment in accordance with the ZÄApprO. 

Practical skills imparted during undergraduate dental education are in addition to cognitive skills also part of the oral-practical state exams. This is also represented in the NKLZ [http://www.nklz.de/kataloge/nklz/lernziel/uebersicht] that mostly aims for competency level 3b (independent and adequate treatment with awareness of consequences) in both basic dental competency as well as professional competency.

## 3. Future Joint Fields of Activity and Research

Despite the fact that the relaying of practical medical skills within the scope of skills labs has so far formed the primary basis for the GMA practical skills committee’s work and thus provides fewer direct connection points to the specific situation regarding dentistry, there nevertheless are entry points for a future cooperation against the background of NKLM and NKLZ and advancement in dental simulators from the GMA dental committee’s point of view: Interdisciplinary and disease- or patient-related taught practical skills on competence level 3a and b require the establishment of workplace based exams in an actual clinical treatment environment – this lends itself to an exchange and collaboration with the GMA committees for practical skills and assessment. Furthermore, in addition to traditional dental simulators, virtual 3-D-patients based on haptic technology become more and more available, providing the means for practising practical skills (e.g. preparation of teeth) in the context of individual patient history, including aspects of diagnostic and differential therapy [2]. Here dentistry might possibly benefit from insights gained in medical skill labs when it comes to the question of how far practical skills and diagnostic abilities practised through self-study or under the instruction of peers could increase competence concerning treatment of actual patients.

## Notes

Authors have written this report in their capacity as vice chairperson^1^ und chairperson^2^ of the GMA committee for dentistry. 

## Competing interests

The authors declare that they have no competing interests.
